# Hyper-realistic Face Masks in a Live Passport-Checking Task

**DOI:** 10.1177/0301006620904614

**Published:** 2020-02-03

**Authors:** David J. Robertson, Jet G. Sanders, Alice Towler, Robin S. S. Kramer, Josh Spowage, Ailish Byrne, A. Mike Burton, Rob Jenkins

**Affiliations:** Department of Psychology, University of York, UK; School of Psychological Sciences and Health, University of Strathclyde, UK; Department of Psychology, University of York, UK; Department of Psychology and Behavioural Sciences, London School of Economics and Political Science, UK; Department of Psychology, University of York, UK; School of Psychology, University of New South Wales, Australia; Department of Psychology, University of York, UK; School of Psychology, University of Lincoln, UK; Department of Psychology, University of York, UK; Division of Psychology and Language Sciences, University College London, UK; Department of Psychology, University of York, UK; Department of Psychology, Edge Hill University, UK; Department of Psychology, University of York, UK

**Keywords:** masks, silicone, realistic, face perception, face recognition, passports, identification, fraud, deception

## Abstract

Hyper-realistic face masks have been used as disguises in at least one border crossing and in numerous criminal cases. Experimental tests using these masks have shown that viewers accept them as real faces under a range of conditions. Here, we tested mask detection in a live identity verification task. Fifty-four visitors at the London Science Museum viewed a mask wearer at close range (2 m) as part of a mock passport check. They then answered a series of questions designed to assess mask detection, while the masked traveller was still in view. In the identity matching task, 8% of viewers accepted the mask as matching a real photo of someone else, and 82% accepted the match between masked person and masked photo. When asked if there was any reason to detain the traveller, only 13% of viewers mentioned a mask. A further 11% picked disguise from a list of suggested reasons. Even after reading about mask-related fraud, 10% of viewers judged that the traveller was not wearing a mask. Overall, mask detection was poor and was not predicted by unfamiliar face matching performance. We conclude that hyper-realistic face masks could go undetected during live identity checks.

## Introduction

Relying on unfamiliar face recognition to verify identity is an important aspect of national security (Robertson & Burton, 2016). In the context of border control, officials are routinely required to decide whether a traveller’s passport photo matches the traveller’s face. False acceptance in this situation could result in an identity fraudster entering the country. Despite the social and economic investment in face photo ID in security critical situations, matching instances of unfamiliar faces remain highly prone to error (Papesh, 2018; Robertson, 2018; White, Kemp, Jenkins, Matheson, & Burton, 2014). It is also a process that fraudsters wishing to deceive ID checkers actively exploit (Robertson, Kramer, & Burton, 2017; Robertson et al., 2018).

Opportunistic identity fraud relies on the fraudster obtaining photo-ID of someone who looks similar to them. In such cases, fraudsters can increase the likelihood of their deception succeeding by disguising their own face so that it looks more like the face of their victim. Traditional methods of disguise have tended to focus on simple paraphernalia such as glasses and wigs (Dhamecha, Singh, Vatsa, & Kumar, 2014; Kramer & Ritchie, 2016; Righi, Peissig, & Tarr, 2012; Terry, 1994). However, a number of recent criminal cases have raised the profile of a different approach—hyper-realistic silicone masks that completely transform the appearance of the wearer (Sanders et al., 2017; Sanders & Jenkins, 2018).

In one widely cited example, a young Asian man used a hyper-realistic mask to impersonate an elderly Caucasian man whose passport he had stolen. Wearing the mask, the fraudster passed through several identity checks at Hong Kong airport and successfully boarded a flight to Canada. The deception was detected only when he removed the mask during the flight, and a fellow traveller reported the incident to the crew (Zamost, 2010). This example suggests that hyper-realistic face masks can be sufficiently convincing to pass for real faces. Importantly, this appears to be the case even at passport control, where an official’s attention is directly focused on facial image comparison.

Despite the threat posed by this new type of fraud, few experiments have addressed detection of hyper-realistic face masks. Sanders et al. (2017; Experiment 1) asked participants to rate the appearance of 20 face photos on (task irrelevant) social dimensions such as attractiveness. Unbeknownst to the participants, one of these photos showed a person wearing a hyper-realistic mask. Following the rating task, participants were given the opportunity to report this imposter in a series of increasingly leading questions. None of the participants reported the presence of the mask spontaneously or when prompted with a general question about the appearance of the faces. Moreover, only 22% of participants guessed that the face images included a mask when explicitly asked. When shown an array of all the images and asked to pick out the mask, 30% of participants missed the mask, and nearly every real face was singled out as the mask by at least one participant. These findings suggest that the detection of hyper-realistic masks is difficult when comparing photos. Even when the viewer is aware that a mask is present, detection levels remain far from perfect.

**Figure 1.**
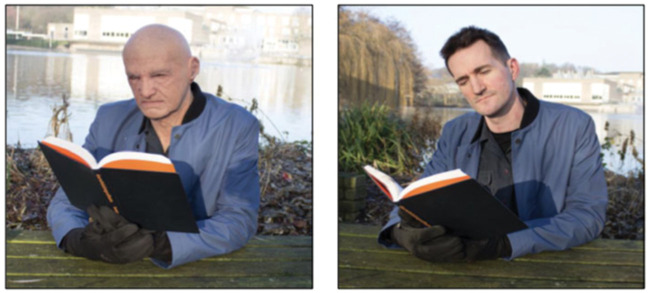
Illustration of live viewing conditions from Sanders et al. (2017; Experiment 3). The images show the confederate (author R. J.) wearing the mask (left) and the confederate’s real face (right). Images reproduced with permission of the authors.*Note:* Please refer to the online version of the article to view the figures in colour.

Sanders et al. (2017; Experiment 3) also examined detection of masks in live viewing. As seen in Figure 1, a mask-wearing confederate sat at a bench on a university campus, and experimenters stopped passers-by to ask them questions about the confederate’s appearance. Respondents viewed the confederate at a distance of 5 m (Near) or 10 m (Far). As with the photographic study, participants were initially asked to rate the individual on social dimensions such as attractiveness. They then turned towards the experimenter (away from the confederate) to answer the open, prompted, and explicit questions concerning mask detection. None of the participants in the Far condition (10 m), and only 6% of those in the Near condition (5 m), reported the presence of a mask in the open or prompted report. For the explicit report question (i.e., was that person wearing a hyper-realistic mask), only 43% of participants reported that the confederate was wearing a mask (detection rates were significantly higher for those viewing from 5 m than 10 m).

To summarise Sanders et al.’s (2017) study, detection of hyper-realistic masks was poor in both photographic viewing and in live viewing. These low detection rates suggest that hyper-realistic masks may provide a viable route to identity fraud. Here, we assess this possibility directly in a mock border control scenario. Our study design extends the preceding work in four important ways. First, we modelled aspects of a border control setting to test whether participants would ever accept a masked imposter as a match for a real passport photo. The context of a passport document has previously been shown to boost acceptance rates in facial image comparison (McCaffery & Burton, 2016). Second, we used concurrent perceptual matching rather than immediate memory when assessing detection. That is, participants completed the image comparison task and the mask detection questions (open, prompted, and explicit) with the mask wearer directly in view. Third, we used a closer viewing distance. Sanders et al. (2017) used *social* viewing distances of 5 m and 10 m, but passport checks are typically carried out at 1 to 2 m (Noyes & Jenkins, 2017; Verhoff, Witzel, Kreutz, & Ramsthaler, 2008). We use a viewing distance of 2 m to capture this applied constraint. Finally, we examined individual differences in face-matching ability. Here, we assess whether those who score highly on the Glasgow Face Matching Test (GFMT; Burton, White, & McNeill, 2010; Robertson, Noyes, Dowsett, Jenkins, & Burton, 2016) are more likely to detect a hyper-realistic face mask. We expected that the gravitas of the passport context, the availability of the masked face during the task, the closer viewing distance, and the high face-matching aptitude of some observers would lead to high detection rates for the mask.

## Methods

### Ethics Statement

This study was approved by the Ethics Committee of the Department of Psychology, University of York and the London Science Museum. All participants provided written informed consent. The participants shown in Figure 3 provided appropriate photographic release.

### Participants

Fifty-four participants (37 female, 17 male) with a mean age of 28 years (*SD* = 7, range = 18–49) volunteered as part of a public engagement event at the London Science Museum. During the experimental debrief, all participants confirmed that they had no prior knowledge that a hyper-realistic mask was being used in this study.

### Design and Procedure

#### Overview

Testing took place on a single evening at the London Science Museum. The study comprised three phases, and all participants completed these phases in the same sequence. In Phase 1, we used the short version of the GFMT to estimate unfamiliar face-matching ability. For Phase 2, participants proceeded to a mock passport control area. The task in this phase was to verify the identity of a traveller (an experimental confederate) by comparing a passport photo to his live appearance. Finally, in Phase 3, participants completed a short questionnaire that was designed to assess detection of the hyper-realistic face mask. Together, these measures allowed us to estimate both the rate of mask detection and the predictive value of face-matching accuracy in this situation. The testing space was divided into three areas—a GFMT testing area, a passport control area, and a debrief area. The layout ensured that participants could not see the traveller before entering the passport control area and could not hear the debrief before entering the debrief area.

### Phase 1: Face-Matching Ability

The short version of the GFMT consists of 40 pairs of unfamiliar face photos presented in a random sequence on a computer screen. In 20 of these pairs, both photos show the same identity. In the remaining 20 pairs, the two photos show different identities. For each pair, the participants’ task is to decide whether the photos show the same person or two different people. Participants’ scores out of 40 are converted to percentage scores for analysis.

### Phase 2: Mock Passport Check

#### Passport photo-to-face matching

**Figure 2.**
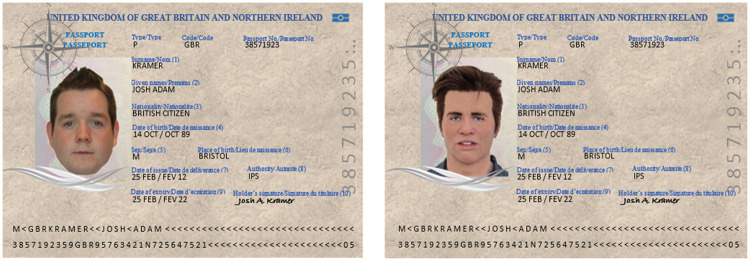
Mock passport check (due to copyright reasons, we cannot show the actual passports used in the study; however, the images that we present here are a close approximation). The left panel shows a mock passport containing a photo of a real face (due to copyright reasons, we could not show the actual foil identity used in the study). The right panel shows a mock passport containing a photo of the masked confederate. Participants received either a passport containing a photo of the foil identity or of the confederate wearing the mask and were asked to decide whether the face in the passport photo matched the person in front of them (viewing distance 2 m).*Note:* Please refer to the online version of the article to view the figures in colour.

To reinforce the participant’s role as passport checker, and to approximate the real-world visual demands of photo-to-face comparison, we embedded the face photographs in realistic passport documents, as seen in Figure 2 (McCaffery & Burton, 2016). The demographic information (e.g., sex, date of birth) in these documents was the same for match and mismatch images. Pilot testing confirmed that this information was plausible for both the face photos and mask wearer. We created two versions of the mock passport. The first version contained a photo of experimental confederate Josh (author J. S.) wearing the hyper-realistic mask (photo taken 2 weeks before testing). This version allowed us to examine detection of a mask that was presented live and in the ID document. The second version contained a photo of a real person (no mask) whose facial appearance was similar to the mask (i.e., young White male with dark hair). This version models a form of identity fraud in which a fraudster has obtained a mask that resembles the identity in the stolen document. The two versions of the passport were alternated across participants. In each case, the participants’ task was to decide whether the photo in the passport showed Josh or someone else (identity matching).

#### Masked confederate

**Figure 3.**
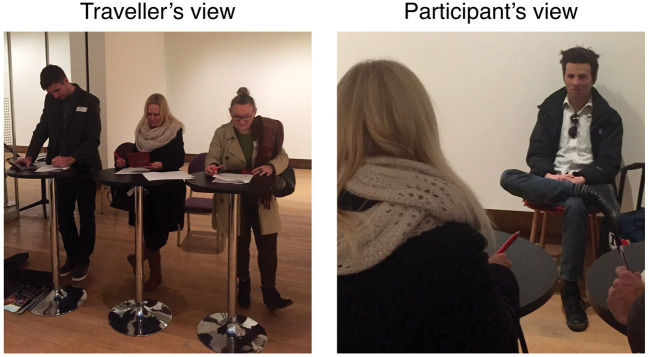
The passport control area showing (left) participants carrying out the mock passport check and (right) masked confederate Josh from the participant’s point of view (2 m viewing distance). Participants shown in Figure 3 provided appropriate photographic release.*Note:* Please refer to the online version of the article to view the figures in colour.

**Figure A1.**
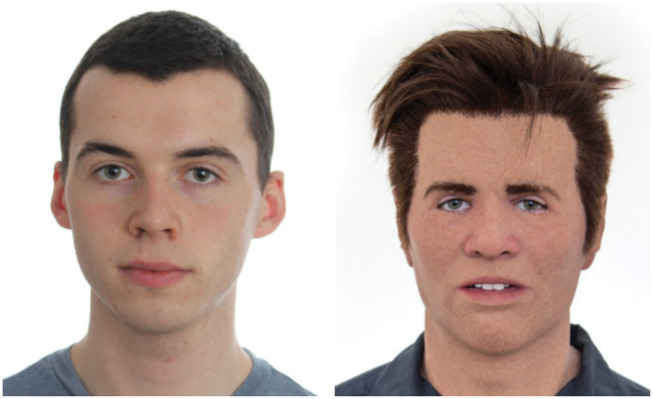
Left image shows the real face of mask wearer (author J. S.) and with the addition of the mask on the right.*Note:* Please refer to the online version of the article to view the figures in colour.

An experimental confederate, Josh (author J. S., real face included in Figure A1), played the role of the traveller. Josh was seated 2 m from the participants’ desk for the duration of testing, as seen in Figure 3. Unbeknownst to the participants, Josh was wearing a hyper-realistic silicone mask (the *Male Model* mask, from Realflesh Masks, Montreal, Quebec). This aspect of the study was not mentioned to participants until debriefing. Participants were instructed that the traveller was returning to the United Kingdom from Spain. Josh was provided with props (e.g., hand luggage with an “I love Barcelona” sticker) to reinforce this cover story. Our main interest was (a) the participant’s response to the identity comparison and (b) whether the participant noticed the mask.

### Phase 3: Mask Detection

Following Sanders et al. (2017), the questionnaire comprised a series of increasingly leading items. The first item (spontaneous detection) allowed participants to report spontaneously that the traveller was wearing a mask (open response). The second item (prompted detection) raised the possibility that the traveller was wearing a disguise (checklist responses). The third item (categorical detection) asked directly whether the traveller was wearing a mask (Yes/No response). The three questions were printed on separate pages so that participants could only advance to the next question after being instructed to do so by the experimenter. The questions were as follows:

#### Spontaneous detection (open response)

Regardless of whether the passport photo shows Josh or not, is there *any other reason* why you would not allow him to enter the United Kingdom?

#### Prompted detection (checklist responses)

Has Josh disguised his appearance (Y/N)? (critical item). Is Josh’s date of birth suspicious (Y/N)? Should Josh’s luggage be searched for drugs (Y/N)? Do you suspect that Josh is carrying more than the 4-litre allowance of wine in his luggage (Y/N)? Josh claims to have been in Spain for a business trip. Is there any reason to believe that this was not the true purpose of his visit (Y/N)? If you have circled “Yes” to any of the previous questions, please briefly explain why in the response box below.

#### Categorical detection (yes/no response)

This workshop runs for more than 3 hours. Half the time Josh will be a regular law-abiding traveller. At other times, Josh is a fraudster and will be wearing a hyper-realistic face mask. He does this to make himself look more like the person whose passport he has stolen. Is Josh wearing a hyper-realistic mask right now? (Circle “Yes” or “No” and briefly describe why you have made that choice in the response box below).

## Results

### Face-Matching Ability

Mean accuracy on the GFMT was 82% (*SD* = 12%, range = 50%–100%). Importantly, as this test was administered to the general public in a museum setting, this distribution was very similar to published norms (Burton et al., 2010; *N* = 194; *M* = 81%, *SD* = 10%, range = 50%–100%).

### Mock Passport Check

We analysed responses in the passport check separately for the two versions of the passport document. For the version containing a photo of Josh wearing the mask, the acceptance rate was 82%. For the version containing a photo of someone else (no mask), the acceptance rate was 8%.

### Mask Detection

Mask detection data are summarised in Table 1. Only 13% of participants spontaneously reported that the traveller was wearing a mask. Of the remaining participants, a further 11% indicated when prompted that the traveller had disguised his appearance.

**Table 1.** Proportion (%) of Participants Who Detected (Yes) or Did Not Detect (No) the Mask at Each Detection Stage.

Detection stage	Yes (%)	No (%)
Spontaneous detection	13	87
Prompted detection	11	89
Categorical detection	90	10

As can be seen from Table 2, viewers were more likely to query the purpose of the traveller’s trip or the contents of his luggage than to suspect that he was disguised. Even when we drew attention to the issue of mask fraud and informed participants that the traveller may be wearing a mask (categorical detection), only 90% of participants thought that he was. In other words, 10% of participants judged that Josh was *not* wearing a mask, even while viewing him from a distance of 2 m.

**Table 2.** Proportion (%) of Participants Who Checked Each Reason to Deny the Traveller Entry at the Prompted Detection Stage.

Reason to deny entry	Yes (%)
Disguised appearance	36
Suspicious date of birth	34
Drug check	55
Wine limit	15
Business trip	66

*Note*. Participants were free to check as many or as few reasons as they liked.

### Justification of Responses

**Figure 4.**
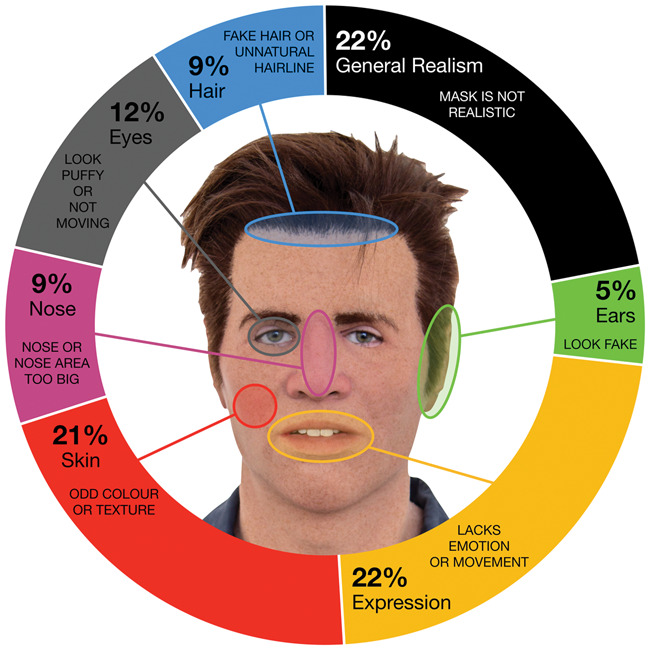
Proportions of written justifications that mentioned each cue (categorical detection task).*Note:* Please refer to the online version of the article to view the figures in colour.

Participants gave a range of reasons for “Yes” responses at the categorical detection stage. Most participants (78%) attributed their response to a specific cue. Figure 4 shows these responses broken down by face region. Unattributed detection accounted for only 22% of responses.

### Individual Differences

To test whether unfamiliar face-matching ability was associated with mask detection, we compared GFMT scores for participants who detected the mask at spontaneous or prompted report (*N* = 12, *M* = 83%, *SD* = 10%, range = 68%–98%) and those who did not (*N* = 42, *M* = 81%, *SD* = 12%, range = 50%–100%). A between-subjects *t* test revealed no significant difference between these subgroups either for overall GFMT scores or scores on the match and mismatch conditions separately (all *t*s* *<* *1). GFMT scores for participants who failed to detect the mask in the categorical (Yes/No) report were also normal (*M* = 85%, *SD* = 7%, range = 75%–90%).

## General Discussion

Previous research by Sanders et al. (2017) found that detection rates for hyper-realistic masks were remarkably low. In that study, participants relied on immediate memory of the masked confederate from 5 or 10 m. In contrast, we allowed participants to view the mask wearer throughout testing, and from the shorter distance of just 2 m, similar to passport control conditions (Noyes & Jenkins, 2017). These viewing conditions are much more conducive to mask detection, compared with previous work. Nonetheless, our findings follow a very similar pattern. Only 13% of participants detected the mask during spontaneous report, and from the remaining participants, just 11% detected the mask at prompted report. Even when explicitly asked whether the traveller was wearing a mask, 10% of viewers judged that he was not. Moreover, participants accepted the face of a mask wearer as matching a photo of another person 8% of the time (cf. Zamost, 2010). These findings suggest that a hyper-realistic silicone mask can pass for a real face, even when viewers are aware that it could be a mask, and even when their viewing time is not restricted.

Interestingly, participants singled out various aspects of facial appearance to explain their judgement (at the explicit question stage) that the traveller was wearing a mask. This wide range of justifications suggests that there may be no single cue that gave the mask away. A recent analysis by Sanders and Jenkins (2018) found that the most reliable differences between photos of real faces and photos of hyper-realistic masks were in the eye region and that viewers who classified the photos accurately used information in that diagnostic area. However, that analysis was based on dozens of trials involving different faces and different masks, whereas the current study involved one-shot decisions to a single mask wearer. Moreover, Sanders and Jenkins (2018) did not ask participants to explain their classification decisions. It seems entirely plausible that their participants were unaware of their reliance on the eye region. Previous studies have shown that insight into one’s own decision-making is generally limited and that participants often rationalise their own decisions post hoc (Nisbett & Wilson, 1977). This includes decisions concerning face identification (Sauerland et al., 2016). Either way, we found little evidence in this task that successful mask detection could be attributed to any particular facial cue.

Although the participants in this study were members of the general public, it is not clear that professionals whose work involves face viewing would perform any better (see Zamost, 2010 for a real-world example). Previous studies have shown that professional training and experience confer no discernable advantage in face identification tasks (Papesh, 2018; White et al., 2014). While recent research has focused on the selection of individuals who naturally excel at such tasks (Bobak, Dowsett, & Bate, 2016; Bobak, Hancock, & Bate, 2016; Davis, Lander, Evans, & Jansari, 2016), our findings did not show that greater GFMT scores were associated with earlier mask detection, and scores for those who did not detect the mask at all were within the normal range. The suggestion here is that face identification and mask detection may be separable problems. Any relation between them could be clarified by comparing performance distributions on the two tasks.

Our previous studies on this topic have tested many different masks worn by many different people. That approach allowed us to generalise our observations across a range of viewing conditions. Here, we took the complementary approach of testing a single mask in a more ecologically valid setting. Our findings provide an existence proof of an artificial face that can withstand direct scrutiny under live viewing conditions and at close range. The existence of such masks presents some interesting challenges for security and crime prevention. For example, in one recent case, criminals used a silicone mask to impersonate a French minister for video calls with business leaders (Schofield, 2019). The criminals were able to defraud businesses of 80 million euros before being stopped. This case raises interesting issues for future research, including impersonation of faces that are familiar to the viewer. Some very recent work has shown that viewers are better able to see through impersonation disguise when they are familiar with the target of impersonation (Noyes & Jenkins, 2019). However, that work did not consider hyper-realistic face masks as disguises. It is possible that a moderate resemblance would be enough to fool a moderately familiar viewer, while a strong resemblance would be required to fool a highly familiar viewer. In the current study, 8% of participants accepted the image of our foil identity as a match to the mask, but this may be an underestimate of acceptance rates. Our foil image was selected from an existing database of face photos as a good match to our mask. However, in a real attempt at fraud, the perpetrator could have a mask created to resemble the face photo in a stolen passport or could select a target who resembles an existing mask (e.g., Schofield, 2019). Either approach could make the resemblance between the mask and passport photo greater than was possible in this study, potentially leading to higher false acceptance rates.

To mitigate human error at passport control, airports across the world have invested in e-Gates (electronic facial recognition technology) that use an algorithm to match a digital image stored on the passport to the passport holder’s face. Despite this investment, such systems are also prone to identification errors (Phillips et al., 2018). It is not clear how they would perform when comparing a passport image to a mask. In principle, e-Gates could be modified to enhance mask detection. For example, infrared imaging could be used to distinguish the thermal signature of a masked face from that of a real face. Given that the mask does not occlude the wearer’s eyes, an iris scan could identify the wearer.

## Conclusions

To conclude, this study extends the findings of Sanders et al. (2017) to an important applied situation. In a mock passport control task, we found that (a) a hyper-realistic mask was often accepted as a match to a stolen passport photo, (b) spontaneous mask detection was remarkably rare, and (c) raising awareness of mask-related fraud did not fully solve this problem. Based on these findings, we conclude that hyper-realistic masks pose an unresolved problem in identity fraud.
